# Theoretical investigation of the thermal decomposition mechanism of ammonium dinitramide (ADN) as a green propellant using DFT methods

**DOI:** 10.1039/d6ra01352j

**Published:** 2026-04-20

**Authors:** Zakaria Harimech, Adil Souagh, Kainaubek Toshtay, Ahmed Bachar, Assia Mabrouk, Seitkhan Azat, Mohammed Salah, Rachid Amrousse

**Affiliations:** a University Chouaib Doukkali, Faculty of Sciences 24000 El Jadida Morocco harimechzakaria@gmail.com amrousse.r@ucd.ac.ma; b Department of Chemistry and Chemical Technology, Al-Farabi Kazakh National University Almaty 050040 Kazakhstan; c Ibnou Zohr University, Faculty of Applied Sciences Ait Melloul 80000 Morocco; d Institute of Mining and Metallurgical, Satbayev University Almaty 050000 Kazakhstan

## Abstract

The demand for satellite maneuvering and reaction control systems (RCS) is increasing, therefore, space agencies are searching for greener, high performance propellant alternatives. One alternative that fits this description is ammonium dinitramide (ADN), which is a promising eco-friendly option to replace the use of hydrazine based monopropellant because of its high density of energy and low toxicity. An investigation into the mechanism for ADN decomposition in the gas phase has been systematically undertaken using density functional theory (DFT). The geometry and frequencies of the possible decomposition products of ADN have been optimized and calculated at the B3LYP/6–311++G(d,p) level; a reaction pathway was developed based upon the investigation of five reaction routes that could lead to the decomposition products using energies of each molecule's transition state and the intrinsic reaction coordinate. Due to the limited thermal stability that ADN possesses at room temperature, the effect of solvents on the thermodynamics of ADN was modeled and studied in a liquid phase, specifically water and methanol, to gain insight into the thermodynamic and kinetic aspects of the ADN decomposition pathway and provide a greater understanding of the performance and stability of ADN to function as a green propellant in next-generation satellite propulsion systems.

## Abstract 2

The demand for satellite maneuvering and reaction control systems (RCS) is increasing, therefore, space agencies are searching for greener, high performance propellant alternatives.

## Graphical abstract

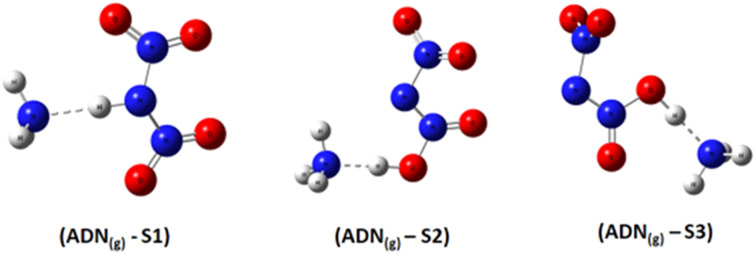

## 1. Introduction

In liquid rocket propellants, ammonium dinitramide (ADN, [NH_4_][N(NO_2_)_2_]) is considered a promising energetic oxidizer to replace traditional compounds such as hydrazine (N_2_H_4_) and ammonium perchlorate (NH_4_ClO_4_), which are expensive and environmentally harmful.^1,2^ The interest in ADN is mainly related to its lower production cost and its reduced environmental impact. Moreover, ADN offers several important advantages, including a less toxic synthesis route, the use of inexpensive and readily available raw materials, a simple and safe preparation process, and good quality of the final product.^3–5^ For example, Suzuki *et al.*^6^ patented a method in which ADN crystals are obtained by reacting urea nitrate and sulfuric acid with a nitrating agent such as nitronium tetrafluoroborate, followed by treatment with gaseous ammonia and filtration. It exhibits a high formation of enthalpy, higher specific impulse—similar or better than hydrazine-based propellants and a superior burning rate.^7,8^ ADN has a 1.81 g cm^−3^ density at 25 °C, more than 25% of oxygen balance, and a 259 seconds specific impulse. Importantly, the decomposition of ADN yields safe and chlorine-free products.^9–12^ Studies have exposed that the main decomposition products include N_2_, H_2_O and O_2_.^10^ The decomposition pathway is initiated by dissociation into dinitraminic acid (HDN – HN(NO_2_)_2_) and ammonia NH_3_, accompanied by exothermic HDN decomposition into nitrous oxide N_2_O and nitric acid HNO_3_.^13^ Other major species reported are N_2_O, H_2_O, HNO_3_, and the recombination product ammonium nitrate (AN – (NH_4_NO_3_).^14^ Limited species like NO, O_2_ and N_2_ have been identified during real-time mass spectrometry (MS) of ADN thermal decomposition.

Many researchers have examined the global decomposition mechanisms of gaseous-phase ADN to clarify the reaction pathways and staged thermodynamic analysis.^13,15–17^ In the present work, we study the elementary ADN decomposition mechanisms in the gas phase using density functional theory (DFT) calculations. Additionally, the effects of solvent and temperature on the reaction mechanism are studied.

## 2. Computational methods

Many researchers have examined the global decomposition mechanisms of gaseous-phase ADN to clarify the reaction pathways and staged thermodynamic analysis.^13,15–17^ In the present work, we study the global ADN decomposition mechanisms in the gas phase using density functional theory (DFT) calculations. Additionally, the effects of solvent and temperature on the reaction mechanism are studied. The Gaussian 09 software package was applied to carry out all quantum chemical simulations. DFT^18,19^ calculations were conducted to explain the thermal decomposition mechanism of ADN. The functional B3LYP coupled with basis set 6–311++G(d,p), was used for geometry optimizations of all stationary points such as reactants, intermediates, transition states, and products. In fact, B3LYP functional with Grimme's D3 dispersion correction and the 6–311++G(d,p) basis set, which offers a dependable balance between computational cost and accuracy for predicting molecular geometries, electronic structure, and non-covalent interactions, were used for all molecular modeling computations. Vibration frequency calculations were performed at the same theoretical level in order to characterize the nature of the stationary points. The absence of imaginary frequencies confirms that the optimized geometries correspond to energy minima, while the transition states^20^ are distinguished by the presence of a single imaginary frequency associated with the reaction coordinate. Transition states were initially located using the QST2 method and subsequently verified by intrinsic reaction coordinate (IRC) calculations to ensure that each transition state properly connects the corresponding reactants and products along the minimum energy path. Thermodynamic parameters, including Gibbs free energies (Δ*G*) and energy barriers *E*_a_, were obtained from the frequency calculations by invoking standard statistical thermodynamics. To study the effect of temperature on the decomposition process, thermodynamic properties were calculated at 298 K, 393 K, and 553 K at a constant pressure of 1 atm.

In order to investigate these solvent effects, the polarizable continuum model (PCM) was used to select water and methanol^21^ as polar solvents. By displaying changes in Gibbs free energy and activation energies, our computation model enabled us to precisely evaluate the impact of solvent addition on the stability of reactants, intermediates, transition states, and products. We were more capable to comprehend the initial and total degradation pathways of ADN under various thermal circumstances thanks to the findings of these two investigations in the gas phase and solvent phase.

## 3. Results

**Fig. 1:**
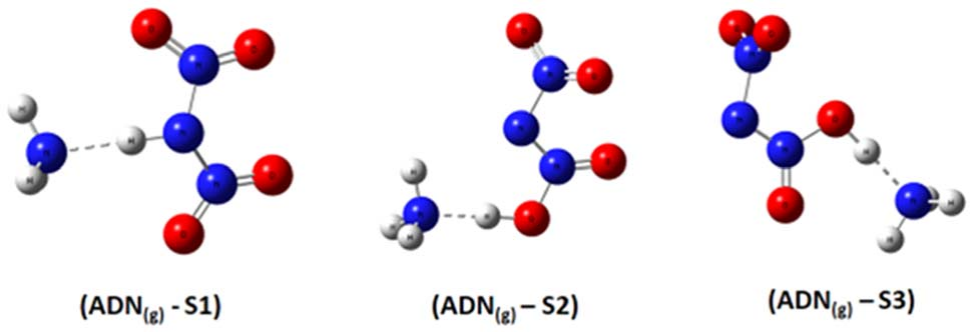
Optimized structures of ADN_(g)_-S1, S2, and S3 by dft (b3lyp-d3/6–311g(d,p)).

Based on DFT calculations using the B3LYP-D3/6–311(d,p) method, we obtained the optimized geometries of three ADN structures, S1, S2, and S3, illustrated in Fig. 1. It was clear that the central nitrogen atom N is the most favorable site for bonding with the hydrogen atom of the NH_4_^+^ groups, corresponding to the ADN_(g)_-S1 structure (Table 1).

**Table 1:** Comparison of structural and energetic properties of ADN(g)-S1, S2, S3 and HDN-S1, S2, S3 derived from DFT/B3LYP-D3/6–311G(d,p) calculations

Species	Bond lengths (Å)	Angles (°)	Energy (kcal mol^−1^)
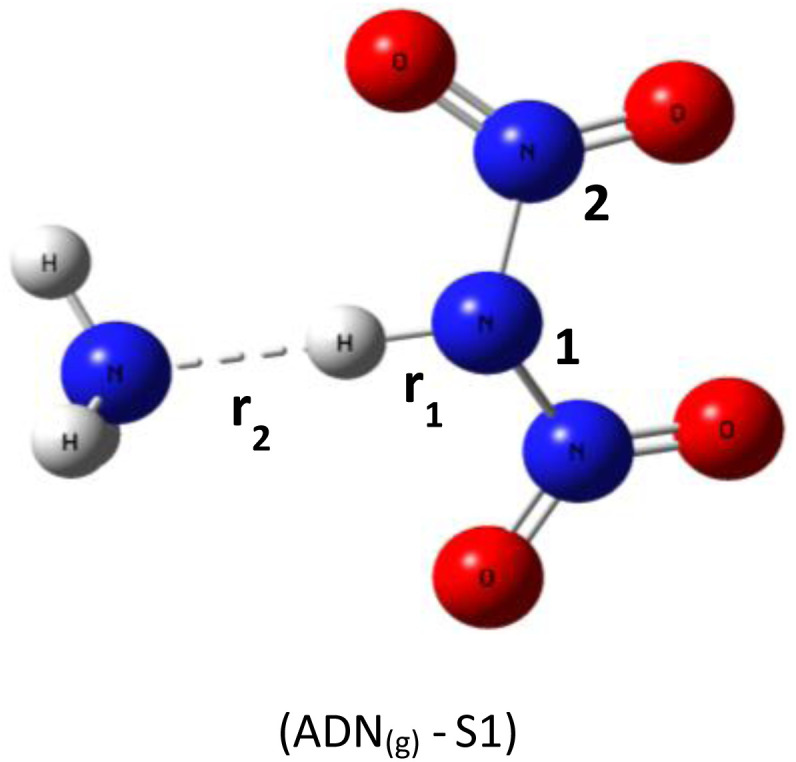	*r*_1_: 1.06 *r*_2_: 1.74	H–N_1_–N_2_: 110.3°	−327 550.56
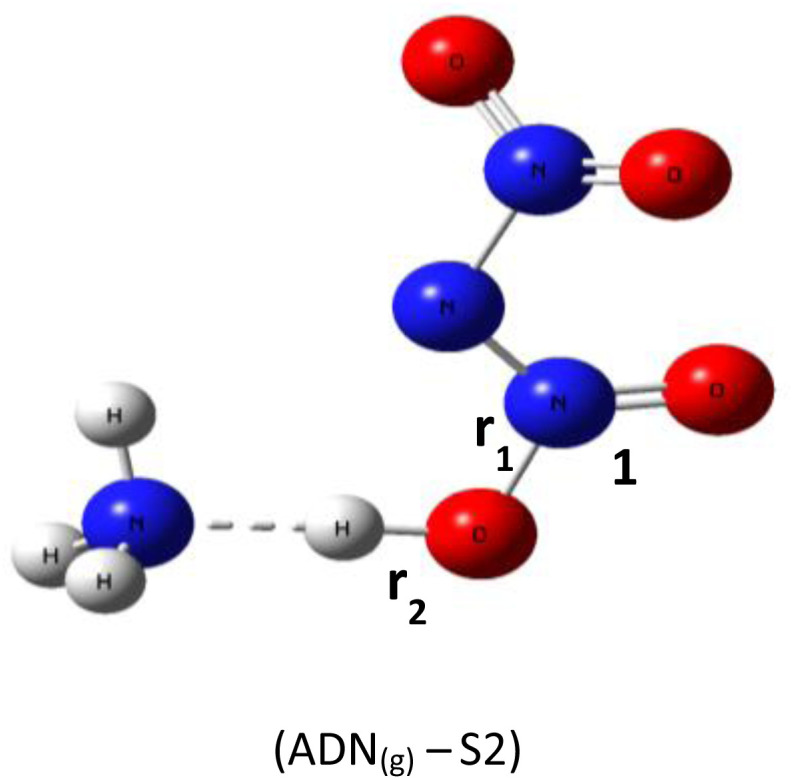	*r*_1_: 1.05 *r*_2_: 1.56	H–O–N_1_: 108.8°	−327 452.68
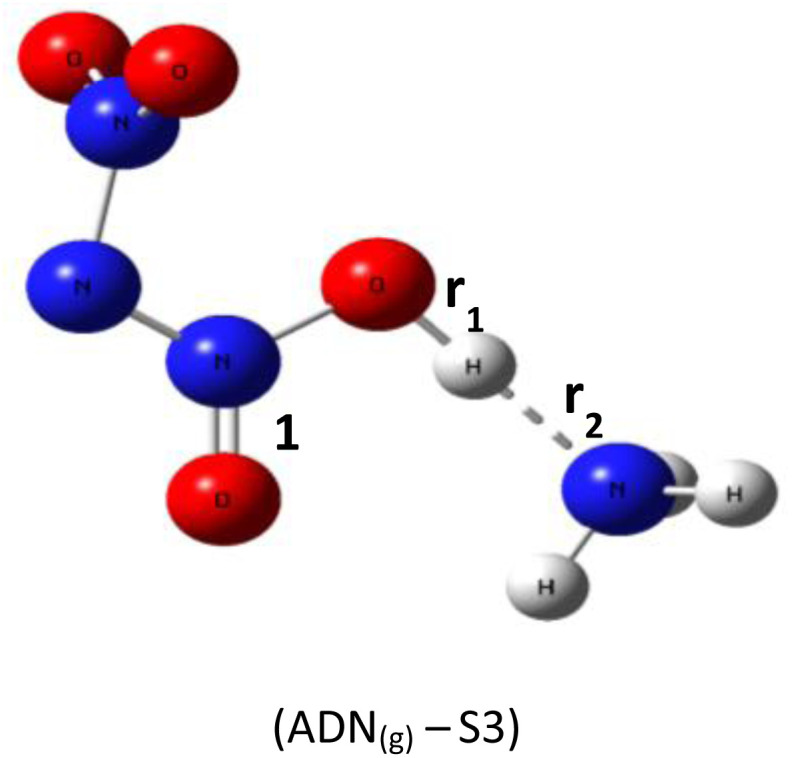	*r*_1_: 1.04 *r*_2_: 1.57	H–O–N_1_: 106.1°	−327 546.16
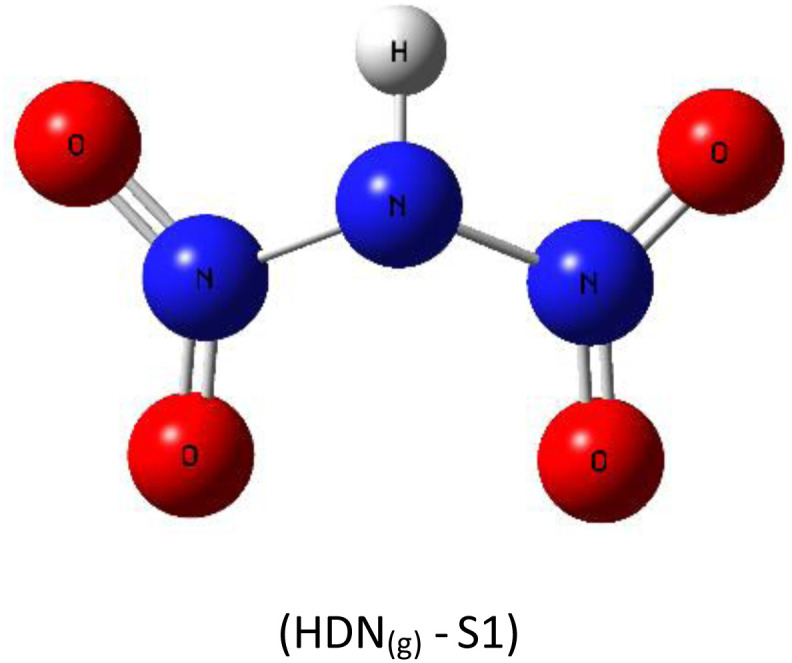	H–N: 1.02	H–N–N: 107.3°	−292 174.70
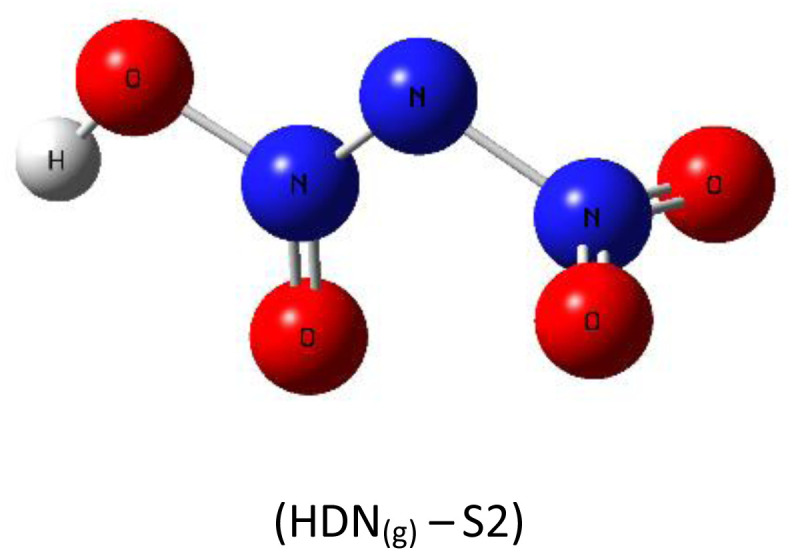	H–O: 0.97	H–O–N: 101.9°	−292 168.42
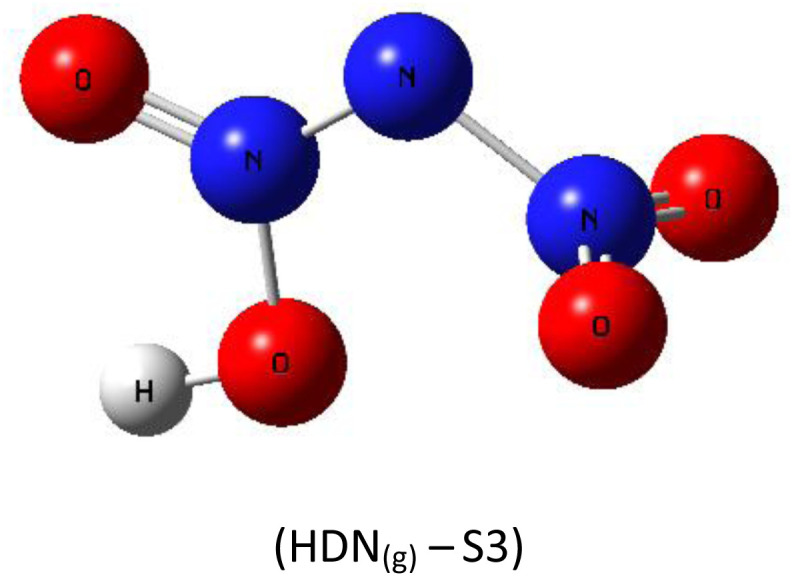	H–O: 0.98	H–O–N: 101.6°	−292 080.42

The relative total energies (kcal mol^−1^) establish the following stability order:*E*_(ADN(g)-S1)_ = −327 550.7 < *E*_(ADN(g)-S3)_ = −327 546.2 < *E*_(ADN(g)-S2)_ = −327 452.7

The lack of imaginary frequencies in the vibrational spectrum of ADN_(g)_-S1 confirms its characterization as a potential energy minimum, thus affirming its thermodynamic stability. This configuration corresponds to the global energy minimum for ADN, establishing it the most stable isomer and the structure chosen for all further investigations. These computational outcomes align with prior theoretical work, such as the study by Wang and colleagues,^15^ which examined the potential energy surface for ADN decomposition involving NH_3_ release. Their research, which computed the system energy along specific reaction coordinates, similarly reported the enhanced stability of the ADN_(g)_-S1 conformation, with an energy advantage of roughly 3 to 4 kcal mol^−1^ relative to other configurations.

**Fig. 2:**
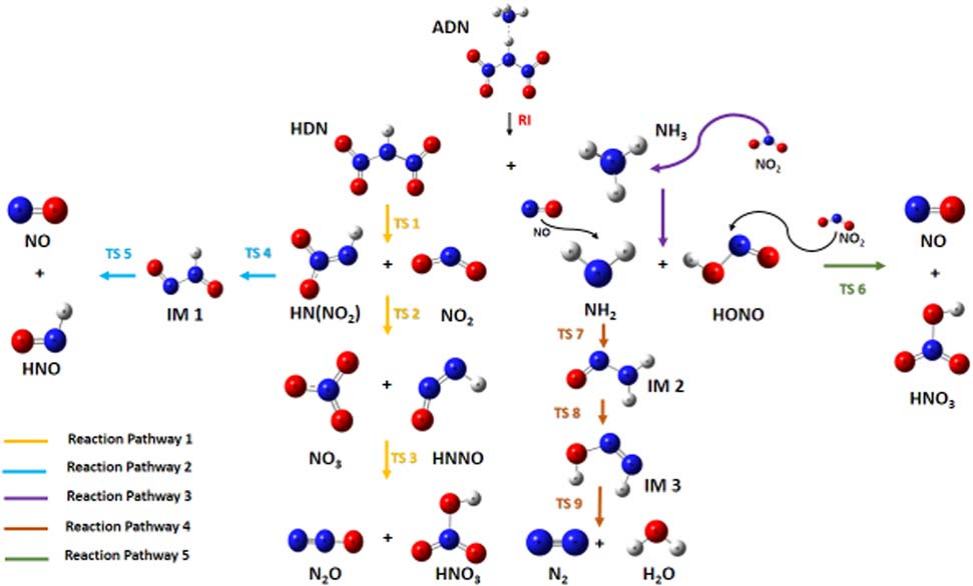
Reaction mechanism for the decomposition of ADN.

In the present paper, our study indicates that the ADN initial decomposition proceeds directly to form HDN and NH_3_, without the intermediate formation of an ammonium ion, as detailed in our proposed mechanism Fig. 2. This dissociation pathway aligns with the work of Korobeinichev *et al.*,^22^ who investigated this process using time-of-flight (TOF) and quadrupole mass spectrometry at atmospheric and reduced pressures (10^−6^, 6, 100 torr) across a temperature range of 80 to 300 °C.

**Fig. 3:**
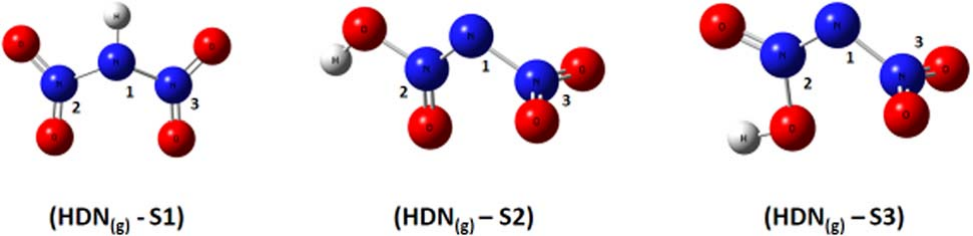
Optimized structures of HDN_(g)_-S1, S2, and S3 by dft (b3lyp-d3/6–311g(d,p)).

A subsequent theoretical investigation, employing an identical computational methodology to that used for ADN, was conducted on HDN to determine the preferred hydrogen atom position following the initial decomposition and to assess whether it remains analogous to the previous configuration. The results for the three candidate structures HDN_(g)_-S1, HDN_(g)_-S2, and HDN_(g)_-S3 Fig. 3 are illustrated in Table 1. According to the statistics, the N1 atom is the most advantageous hydrogen-binding site, providing HDN with the lowest energy and highest stability. The calculated electronic energies are as follows:*E*_(HDN(g)-S1)_ = −292 174.7 < *E*_(HDN(g)-S2)_ = −292 168.4 < *E*_(HDN(g)-S3)_ = −292 080.4 kcal mol^−1^.

These findings are consistent with previous Politzer *et al.*^23^ work, who performed geometry optimizations on the three HDN isomers using various computational levels (HF/6–31G//HF/6–31G, MP2/6–31G//MP2/6–31G, and DF GGA/DZVPP//MP2/6–31G*). They also found that the hydrogen atom bound to the core nitrogen (H–N) is the most stable configuration.

The primary products identified from the thermal decomposition include HNO_3_, H_2_O, N_2_, N_2_O, NO, and NO_2_, among others. Several experimental studies on gas-phase ADN decomposition have confirmed the evolution of these species. For instance, Izato *et al.*^24^ analyzed the gas evolution kinetics and thermal decomposition ADN behavior by thermogravimetry-differential thermal analysis coupled with mass spectrometry and infrared spectroscopy (TG-DTA-MS-IR). The primary released gases were identified as H_2_O (IR absorbance: 4000–3500 and 1800–1400 cm^−1^; MS signals: *m*/*z* = 18, 17), N_2_ (MS: *m*/*z* = 28, 14), N_2_O (IR: 3500–3400, 2250–2100, and 1350–1200 cm^−1^; MS: *m*/*z* = 44, 30, 28, 16), and NH_3_ (IR: ∼950 cm^−1^; MS: *m*/*z* = 17, 16). Similarly, FTIR and GC-MS analysis conducted by Wang *et al.*^15^ on the decomposition gases of ADN at 192 °C primarily revealed the presence of N_2_O (with peaks at 3500–3400, 2250–2100, and 1350–1200 cm^−1^), NO_2_ (1650–1550 cm^−1^), H_2_O (4000–3500 and 1800–1400 cm^−1^), and NH_3_ (750–950 cm^−1^). Furthermore, gaseous decomposition products including HNO_3_, N_2_O, NO_2_, NH_3_, H_2_O, NO, HNO_2_, and N_2_ at 192 °C were also detected by mass spectrometry (MS).

### 3.1. The initiation step in ADN decomposition

The initiation reaction ADN decomposition, described by the global equation:NH_4_N(NO_2_)_2_ → HN(NO_2_)_2_ + NH_3_1

**Fig. 4:**
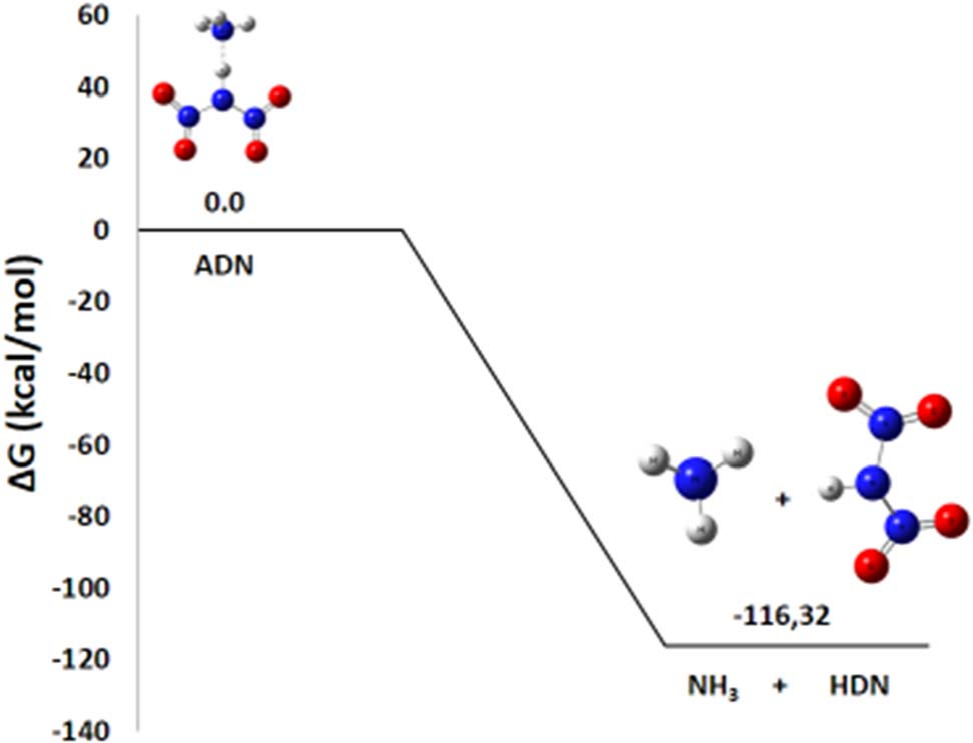
Energy profile of the initial ADN decomposition formation of HDN and NH_3_.

Represents the first separation into ammonia and HDN. This step is considered a common starting point for all subsequent decomposition pathways (1 to 5). The enthalpy change Δ*H*° = −108.2 kcal mol^−1^ is highly negative, indicating an exothermic process that is enthalpically favorable. Regarding entropy, the reaction proceeds from a single molecule to two molecules, increasing the system's disorder, and with Δ*S*° = 27.4 cal mol^−1^ K^−1^, this positive entropic contribution is also favorable. Consequently, the resulting Gibbs free energy change is Δ*G*° (298 K) = −116.3 kcal mol^−1^. This markedly negative value confirms that the dissociation, which requires the rupture of the intramolecular N–H bond, is thermodynamically spontaneous under standard conditions. This observation is in perfect agreement with current theoretical and experimental data that support the fact that ADN split starts with this kind of separation. The optimized geometries for the chemical species involved in this reaction are compiled in Table 1. The energy diagram for ADN initial decomposition Fig. 4 illustrates that the step forming HDN and NH3 corresponds to a Δ*G*° of −116.3 kcal mol^−1^. This suggests that at room temperature (25 °C) and atmospheric pressure (1 atm), the reaction is extremely favorable in the gas phase.

### 3.2. Complete decomposition pathways

#### 3.2.1 Pathway 1: HNO_3_ and N_2_O production route

The pathway 1, describing the HDN decomposition into HNO_3_ and N_2_O (eqn (2)), is also thermodynamically favorable, with a Gibbs free energy change of Δ*G*° = −39.06 kcal mol^−1^.

2 HN(NO_2_)_2_ → HNO_3_ + N_2_O

Nitrous oxide is a commonly observed product in nitrogen-rich energetic materials decomposition. Considering it is well known that HNO_3_ plays an autocatalytic role in the entire decomposition process of ADN, its production is especially significant.^25^

**Fig. 5:**
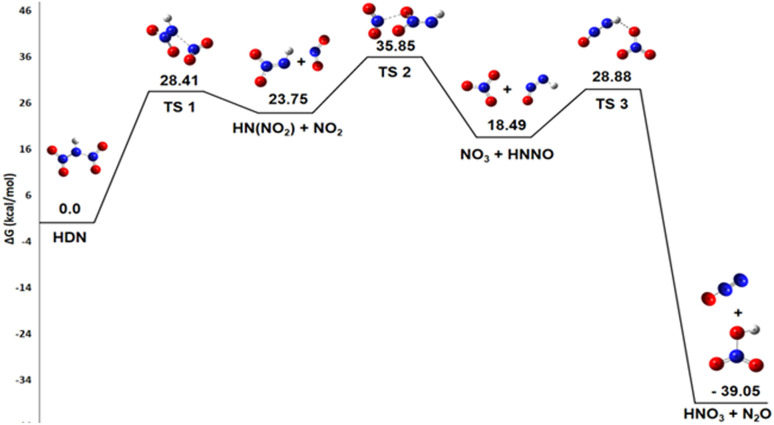
Energy profile of reaction pathway 1: formation of HNO_3_ and N_2_O.

This remark is crucial for understanding the sometimes unpredictable behavior of ADN decomposition, where the presence of HNO_3_ can significantly accelerate the overall reaction rate. Then, the nitric acid product can react with ammonia to reform ammonium dinitramide, a step that is endothermic.^12,26^ There are three transition states involved in the decomposition of HDN to produce HNO_3_ and N_2_O (Fig. 5).

First, the N–N bond breaks to form HN(NO_2_) and NO_2_ radicals *via* a transition state TS1 with an activation energy of 28.41 kcal mol^−1^. For this initial dissociation, the enthalpy change Δ*H*° = +35.4 kcal mol^−1^ is positive, indicating an endothermic and enthalpically unfavorable process requiring energy input. However, the reaction proceeds from one molecule to two radical species, increasing disorder and yielding a positive entropy change Δ*S*° = 45.3 cal mol^−1^ K^−1^, which is entropically favorable. The resulting Gibbs free energy Δ*G*° = +21.9 kcal mol^−1^ is positive, confirming that this step is non-spontaneous under standard conditions at 298 K and would require an energy input to occur. Second, the terminal oxygen atom of HN(NO_2_) (acting as a nucleophile) attacks the nitrogen of NO_2_ (the electrophile), causing the oxygen to migrate to NO_2_ and forming NO_3_ by breaking the N–O bond of the HN(NO_2_) compound. This rearrangement is represented by transition state TS2 with *E*_a_ of 35.85 kcal mol^−1^. For this step, HN(NO_2_) + NO_2_ → NO_3_ + HNNO, the enthalpy change Δ*H*° is nearly zero (+0.1 kcal mol^−1^), indicating a practically athermic, very slightly endothermic reaction. The entropy change Δ*S*° = +2.6 cal mol^−1^ K^−1^ shows a slight increase in disorder, leading to a negative Gibbs free energy Δ*G*° = −0.72 kcal mol^−1^. Although small, this negative value indicates thermodynamic spontaneity under standard conditions, suggesting an equilibrium slightly shifted towards the products. Finally, an oxygen atom from the nascent NO_3_ group attacks the hydrogen atom of the residual HNNO fragment, leading to its detachment as HNO_3_ while the remaining fragment releases N_2_O. This process uses an activation energy of 28.88 kcal mol^−1^ to move through transition state TS3.

For this final step, NO_3_ + HNNO → HNO_3_ + N_2_O, the enthalpy change Δ*H*° = −75.0 kcal mol^−1^ is highly negative, indicating a strongly exothermic and enthalpically favorable process. Although the reaction conserves the number of molecules and the entropy change is slightly negative (Δ*S*° = −22.1 cal mol^−1^ K^−1^), the resulting Gibbs free energy Δ*G*° = −68.4 kcal mol^−1^ is strongly negative. This confirms that the reaction is thermodynamically spontaneous under standard conditions, with the enthalpic contribution largely dominating the process.

#### 3.2.2 Pathway 2: HNO and NO production route

Despite being less exothermic (Δ*G*° = −13.28 kcal mol^−1^), Pathway 2, which splits down HN(NO_2_) into HNO and NO, is still thermodynamically advantageous. One extremely reactive radical that can take part in additional propagation reactions within the larger mechanism is nitric oxide NO.

The pathway first involves the isomerization of HN(NO_2_) into an intermediate (IM1 – HONNO). For this isomerization reaction, the enthalpy change Δ*H*° = −20.5 kcal mol^−1^ is negative, indicating an exothermic and enthalpically favorable process. The reaction conserves the number of molecules, and with an entropy change Δ*S*° = +1.4 cal mol^−1^ K^−1^, there is a slight increase in disorder. The resulting Gibbs free energy Δ*G*° = −20.9 kcal mol^−1^ is negative, confirming that the isomerization is thermodynamically spontaneous under standard conditions. IM1 is therefore thermodynamically more stable than HN(NO_2_). However, the subsequent dissociation of IM1 into HNO and NO is less favorable at room temperature. For the reaction IM1 → HNO + NO, the enthalpy change Δ*H*° = +11.9 kcal mol^−1^ is positive, indicating an endothermic process requiring energy input to break a bond. Notably, this reaction proceeds from one molecule to two radical species, considerably increasing disorder with a highly favorable entropy change Δ*S*° = +35.7 cal mol^−1^ K^−1^. The resulting Gibbs free energy Δ*G*° = +1.25 kcal mol^−1^ is slightly positive, showing that the reaction is not spontaneous under standard conditions at 298 K. However, due to the strong entropic contribution, it becomes spontaneous at higher temperatures.

**Fig. 6:**
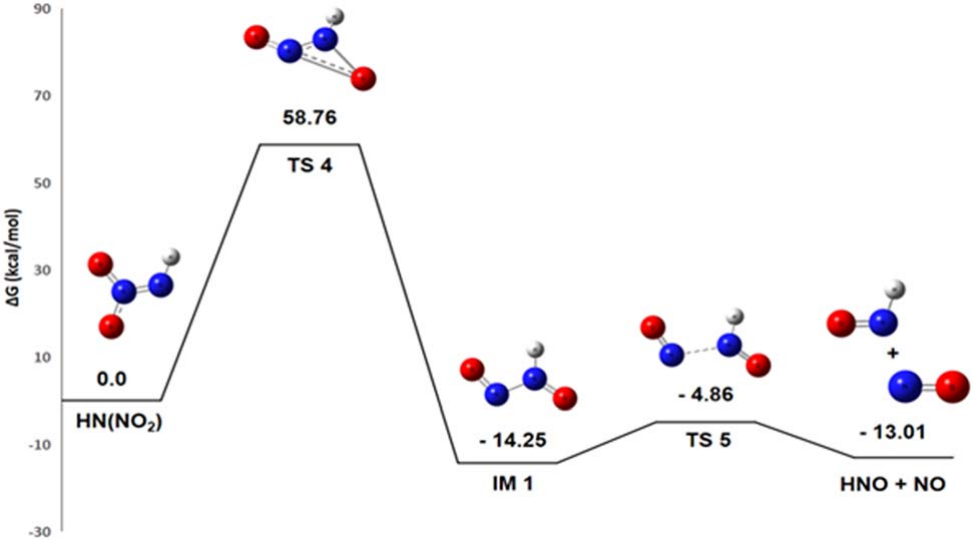
Energy profile of reaction pathway 2: formation of HNO and NO.

The energy diagram in Fig. 6 indicates that the creation of IM1 at ambient temperature in a gaseous state involves a high energy barrier of 58.76 kcal mol^−1^. The subsequent dissociation of this intermediate into HNO and NO is facilitated by the TS5 transition state, which has a lower activation energy of 8.15 kcal mol^−1^. HNO is extremely unstable due to its unfavorable electronic structure. The nitrogen atom in HNO has an unpaired electron, making it a highly reactive radical species. The H–N=O arrangement keeps nitrogen in an unfilled electronic state, which promotes disproportionation. Consequently, HNO is oriented toward a disproportionation reaction (eqn (3), Pathway 2′) with a calculated Δ*G*° of −88.82 kcal mol^−1^, which comes from the expression:

3 2HNO → N_2_O + H_2_O

4 Δ*G*(Pathway 2′) = *E*(H_2_O) + *E*(N_2_O) − 2 × *E*(HNO)

This reaction releases a substantial amount of heat (Δ*G*° < 0), meaning that the products (N_2_O and H_2_O) are significantly more stable than the reactants. This observation reveals that HNO is a transient and unstable species that rapidly transforms into more stable products, thereby contributing significantly to energy release and N_2_O generation.

#### 3.2.3 Pathway 3: NH_2_ and HONO production route

**Fig. 7:**
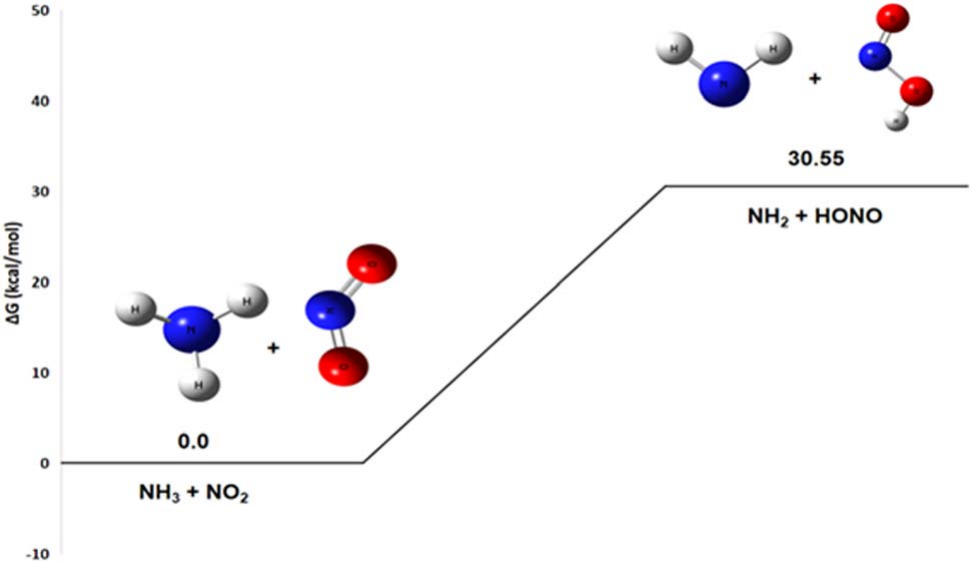
Energy profile of reaction pathway 3: formation of NH_2_ and HONO.

The energy diagram shown in Fig. 7 for the reaction of ammonia with nitrogen dioxide reveals a change in Gibbs free energy of +30.6 kcal mol^−1^. A detailed thermodynamic analysis provides insight into this unfavorable energetics. For the hydrogen abstraction reaction NH_3_ + NO_2_ → HONO + NH_2_, the enthalpy change Δ*H*° = +30.3 kcal mol^−1^ is positive, indicating an endothermic process that is enthalpically unfavorable due to the required rupture of the N–H bond in NH_3_. The reaction conserves the number of molecules, and with an entropy change Δ*S*° = −1.0 cal mol^−1^ K^−1^, there is a very slight decrease in disorder. The resulting Gibbs free energy Δ*G*° = +30.6 kcal mol^−1^ is positive, confirming that the reaction is not spontaneous under standard conditions at 298 K.

In summary, the hydrogen abstraction from NH_3_ by NO_2_ to form HONO and NH_2_ is endothermic and slightly entropically unfavorable, resulting in a non-spontaneous process at room temperature. This positive value indicates that the reaction requires a large amount of activation energy. It is likely that this indirect pathway is made possible by the energy released during other steps of the suggested reaction mechanism and that it can be catalyzed by a product, such as the HNO_3_ generated in the first pathway. This suggests that the reaction would require an energy input or a particular reactive environment to occur.

#### 3.2.4 Pathway 4: HONO and NO_2_ reaction

**Fig. 8:**
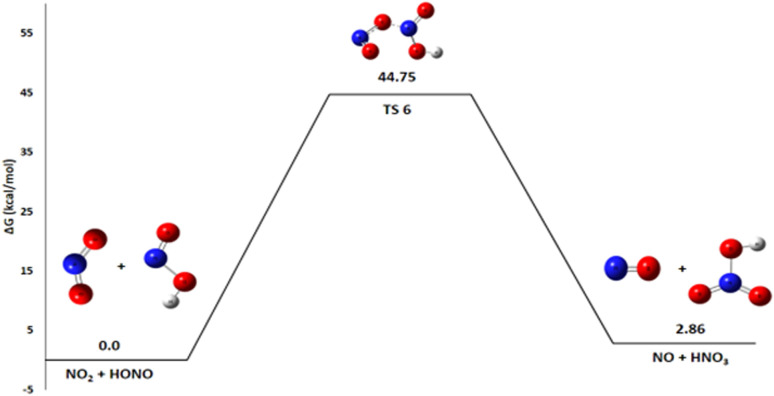
Energy profile of reaction pathway 4: formation of no and HNO_3_.

The reaction in Pathway 4, involving HONO with NO_2_ to form HNO_3_ and NO, exhibits a slightly positive Gibbs free energy (Δ*G*° = +2.86 kcal mol^−1^) as shown in Fig. 8. A detailed thermodynamic analysis provides insight into this unfavorable energetics. The enthalpy change Δ*H*° = +1.26 kcal mol^−1^ is slightly positive, indicating a weakly endothermic process that is enthalpically unfavorable, though to a small extent. The reaction conserves the number of molecules, and with an entropy change Δ*S*° = −5.4 cal mol^−1^ K^−1^, there is a slight decrease in disorder, making it entropically unfavorable as well. The resulting Gibbs free energy Δ*G*° = +2.86 kcal mol^−1^ is positive, confirming that the reaction is not spontaneous under standard conditions at 298 K. In summary, this reaction is very slightly endothermic and entropically unfavorable, leading to a positive Gibbs free energy. This value indicates that the reaction is thermodynamically unfavorable under standard conditions and is not expected to proceed spontaneously to a significant extent. Consequently, this pathway is unlikely to contribute substantially to the primary decomposition mechanism of ADN at room temperature.

#### 3.2.5 Pathway 5: N_2_ and H_2_O production route

**Fig. 9:**
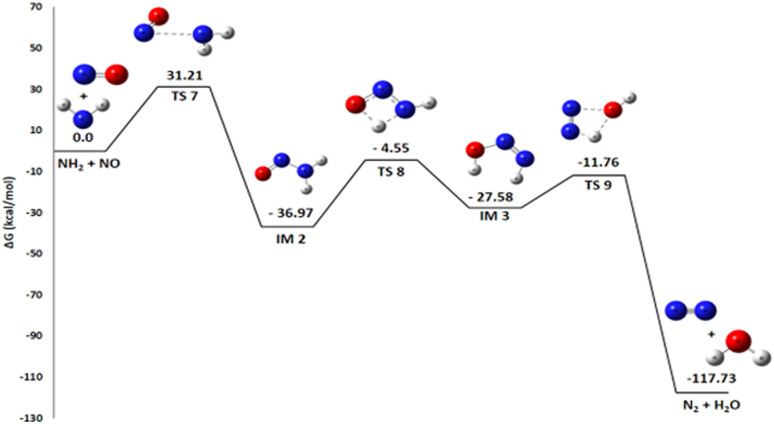
Energy profile of reaction pathway 4: formation of N_2_ and H_2_O.

Pathway 5, illustrating the reaction between NH_2_ and NO to produce N_2_ and H_2_O, is also highly favorable from a thermodynamic point of view Δ*G*° of −117.73 kcal mol^−1^Fig. 9. This reaction is of paramount importance because it is an essential pathway for the formation of nitrogen gas (N_2_), a highly desirable end product in the propellant industry, and contributes significantly to overall energy release. The generation of molecular nitrogen is an essential sign of efficient and complete combustion. The mechanism for the production of N_2_ and H_2_O proceeds through three transition states and two intermediates. A detailed thermodynamic analysis of each elementary step provides insight into the overall favorability. Initially, the nitrogen atom of the NO molecule undergoes a nucleophilic attack on the nitrogen of NH_2_ to form a covalent N–N bond *via* transition state TS7, with an activation energy (*E*_a_) of 31.21 kcal mol^−1^, yielding intermediate IM2 (H_2_NNO). For this recombination reaction NH_2_ + NO → IM2, the enthalpy change Δ*H*° = −47.2 kcal mol^−1^ is strongly negative, indicating a highly exothermic and enthalpically favorable process due to the formation of a strong bond. The reaction proceeds from two radical species to a single molecule, considerably decreasing disorder with an entropy change Δ*S*° = −34.2 cal mol^−1^ K^−1^, which is entropically unfavorable. However, the resulting Gibbs free energy Δ*G*° = −37.0 kcal mol^−1^ is negative, confirming that the reaction is thermodynamically spontaneous under standard conditions, with the enthalpic contribution dominating. The resulting intermediate IM2 subsequently rearranges; the oxygen atom abstracts a hydrogen from the NH_2_ group, and the N–H bond cleaves to form intermediate IM3 *via* transition state TS8 (*E*_a_ = 32.42 kcal mol^−1^). For this isomerization IM2 → IM3, the enthalpy change Δ*H*° = +8.89 kcal mol^−1^ is positive, indicating an endothermic and enthalpically unfavorable process. The reaction conserves the number of molecules, and with an entropy change Δ*S*° = −1.66 cal mol^−1^ K^−1^, there is a slight decrease in disorder. The resulting Gibbs free energy Δ*G*° = +9.39 kcal mol^−1^ is positive, showing that this isomerization is not spontaneous under standard conditions at 298 K. IM2 is therefore thermodynamically more stable than IM3. Finally, the oxygen atom abstracts the remaining hydrogen from the NH group, followed by N–O bond cleavage and product separation *via* transition state TS9, which has an activation energy of 15.82 kcal mol^−1^. For this decomposition IM3 → N_2_ + H_2_O, the enthalpy change Δ*H*° = −73.5 kcal mol^−1^ is strongly negative, indicating a highly exothermic and enthalpically favorable process. The reaction proceeds from one molecule to two species, considerably increasing disorder with a highly favorable entropy change Δ*S*° = +32.4 cal mol^−1^ K^−1^. The resulting Gibbs free energy Δ*G*° = −83.2 kcal mol^−1^ is negative, confirming that the reaction is thermodynamically spontaneous under standard conditions. In summary, the decomposition of intermediate IM3 into N_2_ and H_2_O is strongly exothermic and accompanied by a significant entropy increase, making it thermodynamically very favorable. Despite the non-spontaneous isomerization of IM2 to IM3, the overall pathway from NH_2_ and NO to N_2_ and H_2_O is highly favorable, with the strongly exothermic formation of IM2 and decomposition of IM3 driving the process. This reaction therefore represents a probable termination pathway in the mechanism.

We demonstrate, using DFT calculations performed at 298 K and 1 atm, that the thermal decomposition of ammonium dinitramide initially involves the dissociation of ADN into HN(NO_2_)_2_ and NH3 through N–H bond cleavage, in agreement with previous computational studies.^27,28^ Furthermore, NO_2_ is rapidly generated both from the primary decomposition of ADN and from the subsequent thermal instability of HDN, acting as a key reactive intermediate in several secondary reaction pathways (*e.g.*, Pathways 1, 4, and 5). Although produced in significant initial quantities, its concentration decays rapidly due to highly exothermic secondary reactions, notably with NH_3_ (Pathway 3) to yield N_2_ and H_2_O. The NH_3_ co-produced during the first stage is subsequently fully consumed in the second stage through reactions with NO_2_ or nitroxyl radicals. In contrast to some earlier postulates, the dominant final products are identified as N_2_, H_2_O, and N_2_O,^27–29^ whereas NO and HNO_3_ predominantly appear as transient intermediates. Even though HNO_3_ is generated by thermodynamically favorable processes (Δ*G*° < 0, exothermic), it is not maintained as a stable end product due to its propensity to decompose or engage in radical-driven reactions, including its conversion to ammonium nitrate (NH_4_NO_3_) *via* NH_3_ + HNO_3_ → NH_4_NO_3_. These calculation results are consistent with observations from experiments.^29,30^ They confirm that N_2_O is a predominant product at 298 K, while NO_2_ appears to be temporary in nature.

### 3.3. Temperature effects on ADN thermal decomposition

It is crucial to understand in detail the thermal decomposition of ADN, particularly the impact of temperature on its reaction processes, in order to design safe and efficient propulsion systems. The objective of this chapter is to provide a theoretical and analytical analysis of the influence of temperature on ADN decomposition. We will study the main reaction pathways, the variation in Δ*G*° and *E*_a_ with temperature, and discuss the consequences of using ADN as a substitute for hydrazine. The computer analysis was performed at temperatures relevant to the decomposition and combustion process: 298 K, 393 K, and 553 K.^12,24,29^ All the reaction pathways considered were validated through the identification and characterization of the corresponding transition states.

#### 3.3.1 Initiation step

Our analyses of the decomposition of ADN into dinitric acid and ammonia^24,27^ at various temperatures (298 K, 393 K, and 553 K) reveal that this reaction is thermodynamically spontaneous, and highly exothermic (Δ*G*° < 0, Δ*H*° < 0) across the entire temperature range examined (Table 2).

**Table 2:** Relative energies and activation energies for decomposition reaction pathways at various temperatures and atmospheric pressure^a^

Pathway	Elementary reactions	*T* = 298 K			*T* = 393 K			*T* = 553 K		
	Energies (kcal mol^−1^)	Δ*G*	Δ*H*	*E*_a_	Δ*G*	Δ*H*	*E*_a_	Δ*G*	Δ*H*	*E*_a_
I.S*	ADN → HDN + NH_3_	−116.3	**−108.2**	TS.F *	−118.9	**−108.3**	TS.F *	−123.2	**−108.7**	TS.F *
1	HDN → HN(NO_2_) + NO_2_	21.9	**35.4**	30.0	17.6	**35.5**	30.0	10.3	**35.5**	30.0
	HN(NO_2_) + NO_2_ → NO_3_ + HNNO	−0.7	**0.1**	5.7	−7.8	**4.26**	10.1	−1.5	**0.34**	17.5
	NO_3_ + HNNO → HNO_3_ + N_2_O	−68.5	**−75.0**	11.8	−66.3	**−75.1**	15.6	−62.7	**−75.3**	22.1
2	HN(NO_2_) → HONNO	−20.9	**−20.5**	52.1	−21.0	**−20.4**	51.8	−21.3	**−20.3**	51.2
	HONNO → HNO + NO	1.2	**11.9**	9.4	−2.1	**11.9**	8.9	−7.9	**11.7**	7.9
3	NH_3_ + NO_2_ → HONO + NH_2_	30.6	**30.3**	TS.F *	30.6	**30.4**	TS.F *	30.6	**30.7**	TS.F *
4	HONO + NO_2_ → HNO_3_ + NO	2.9	**1.26**	44.7	3.4	**1.3**	48.3	4.2	**1.3**	54.3
5	NH_2_ + NO → H_2_NNO	−36.9	**−47.2**	31.2	−33.7	**−47.4**	34.4	−28.1	**−47.5**	39.7
	H_2_NNO → HN_2_OH	9.4	**8.89**	32.4	9.6	**8.8**	32.7	9.9	**8.8**	33.2
	HN_2_OH → N_2_ + H_2_O	−83.2	**−73.5**	15.8	−86.3	**−73.2**	15.5	−91.7	**−73.1**	14.9

a TS.F*: TS-free I.S*: Initiation Step.

**Fig. 10:**
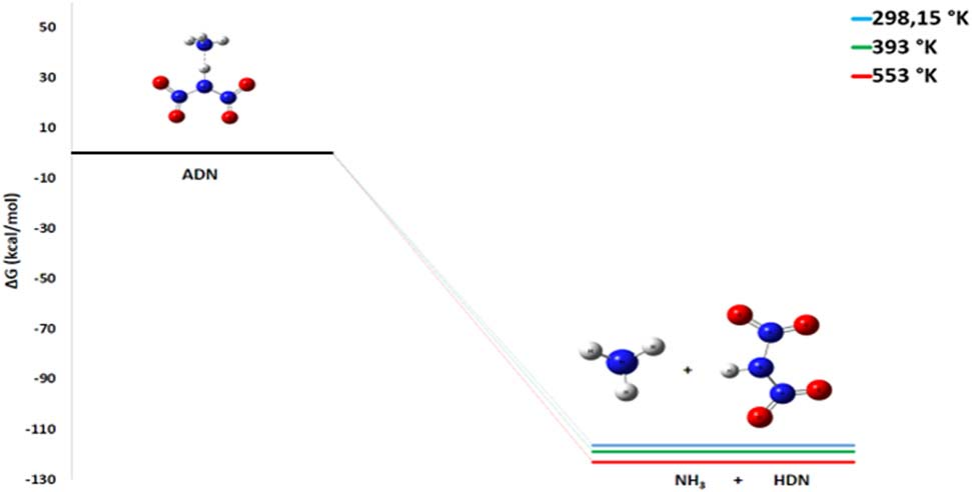
Energy profile of the initiation step at various temperatures and atmospheric pressure.

As the temperature increases, the Δ*G*° value becomes more negative, changing from −116.32 kcal mol^−1^ at 298 K to −123.17 kcal mol^−1^ at 553 K (Fig. 10). A similar trend is observed for the enthalpy change, which slightly varies from −108.2 kcal mol^−1^ at 298 K to −108.7 kcal mol^−1^ at 553 K.

This trend is explained by the influence of the entropic term (−*T*Δ*S*) in the Gibbs equation (Δ*G*° = Δ*H*° − *T*Δ*S*°). The formation of two gaseous molecules from the parent ionic ADN molecule results in a significant increase in disorder (Δ*S* > 0), which makes the reaction increasingly favorable at higher temperatures. The absence of a detectable activation barrier in our calculations aligns with experimental observations of rapid decomposition. As the temperature rises, the production rate of the products (NH_3_ and HDN) produced during the decomposition process increases significantly.

#### 3.3.2 Pathway 1: production of N_2_O and HNO_3_

This most favorable pathway consists of three elementary steps:

5 HN(NO_2_)_2_ → HN(NO_2_) + NO_2_

6 HN(NO_2_) + NO_2_ → NO_3_ + HNNO

7 NO_3_ + HNNO → HNO_3_ + N_2_O

**Fig. 11:**
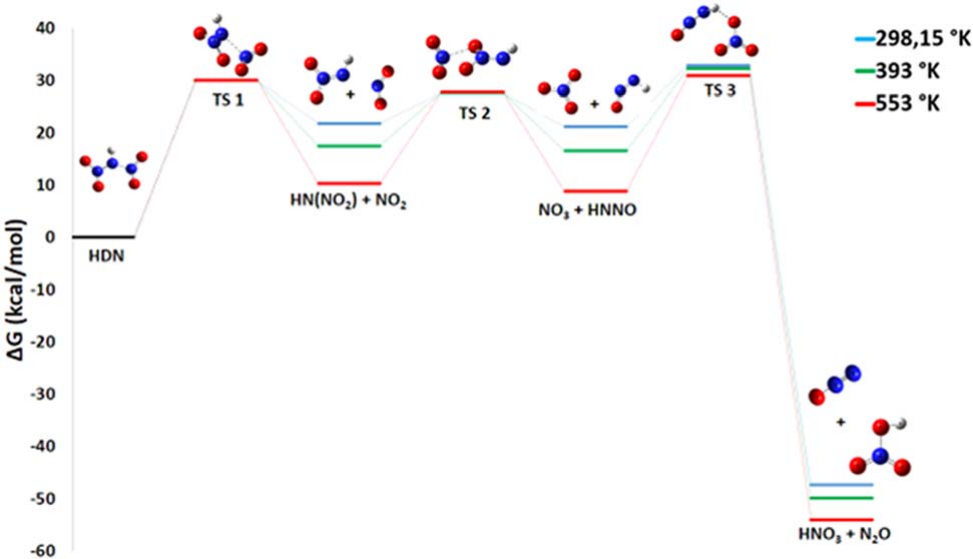
Energy profile of reaction pathway 1 at various temperatures and atmospheric pressure.

The calculated Gibbs free energy changes (Δ*G*) for each elementary reaction reveal a dual effect of increasing temperature. On one hand, the reaction of step 1 becomes more thermodynamically accessible with Δ*G*° and Δ*H*° decreasing significantly (Δ*G*° from +21.9 to +10.3 kcal mol^−1^, Δ*H*° from 35.4 to 35.5 kcal mol^−1^), due to the favorable entropic gain (Δ*S*° > 0) associated with the formation of the liberated radicals. On the other hand, a noticeable increase in the calculated activation energies (*E*_a_) is observed for steps 2 and 3 (Fig. 11). This tendency indicates that the transition states associated with these subsequent reactions exhibit lower entropy, meaning they are more ordered than the corresponding reactants. Such behavior is consistent with associative mechanisms that require a specific and well-defined molecular orientation. Moreover, the final step is highly exergonic and spontaneous, with Δ*G*° values ranging from −68 to −62 kcal mol^−1^ and Δ*H*° values between −75.0 and −75.3 kcal mol^−1^. This strong thermodynamic driving force promotes the substantial formation of N_2_O, a gaseous product that has been consistently observed in experimental investigations.

#### 3.3.3 Pathway 2: production of NO and HNO

This pathway, critical for the generation of gaseous oxidizers, is strongly promoted by increasing temperature. It proceeds *via* two key steps:

8 HN(NO_2_) → HONNO

9 HONNO → HNO + NO

**Fig. 12:**
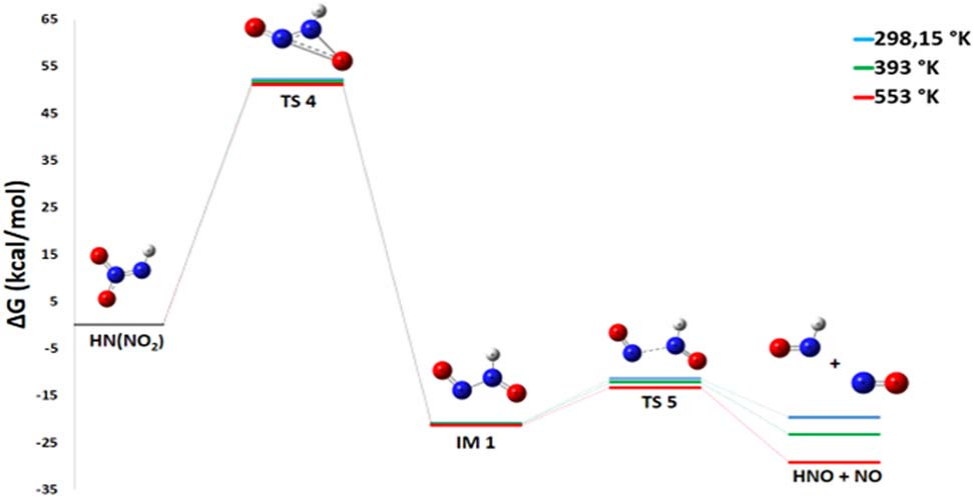
Energy profile of reaction pathway 2 at various temperatures and atmospheric pressure.

For the second step, the activation energy (Fig. 12) and enthalpy changes shows a slight decrease with rising temperature (*E*_a_ from 9.4 to 8.0 kcal mol^−1^, Δ*H*° from 11.9 to 11.7 kcal mol^−1^). More significantly, the Gibbs free energy change (Δ*G*°), which is slightly positive at 298 K, becomes strongly negative at 553 K (−7.9 kcal mol^−1^).

This shift demonstrates that higher temperatures make the reaction not only kinetically faster but also thermodynamically spontaneous. Consequently, high temperature significantly accelerates the production of NO, a key intermediate for subsequent combustion chain reactions.

#### 3.3.4 Pathway 3: production of HONO and NH_2_

Pathway 3 involves the generation of unstable molecules from ammonia and nitrogen dioxide, according to the following reaction:

10 NH_3_ + NO_2_ → HONO + NH_2_

**Fig. 13:**
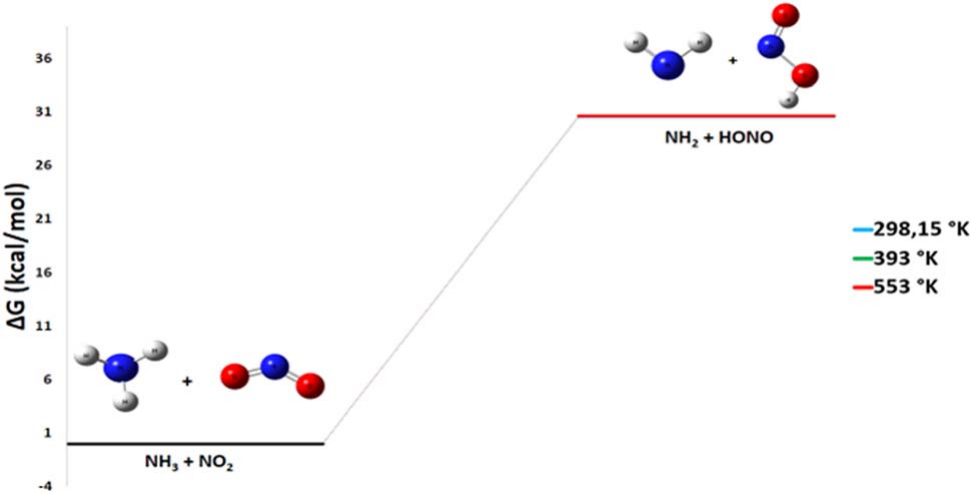
Energy profile of reaction pathway 3 at various temperatures and atmospheric pressure.

This process is strongly endothermic and thermodynamically non-spontaneous, with both the Gibbs free energy change (Δ*G*°) and the enthalpy change (Δ*H*°) calculated to be approximately +30 kcal mol^−1^ across the three investigated temperatures. The reaction remains thermodynamically unfavorable across the entire temperature range investigated (Fig. 13). In practical terms, this pathway does not contribute significantly to the final product distribution, as the substantial energy requirement prevents its efficient initiation under the studied conditions.

#### 3.3.5 Pathway 4: production of HNO_3_ and NO

This pathway describes the formation of nitric acid and nitric oxide *via* the bimolecular reaction:

11 HONO + NO_2_ → HNO_3_ + NO

**Fig. 14:**
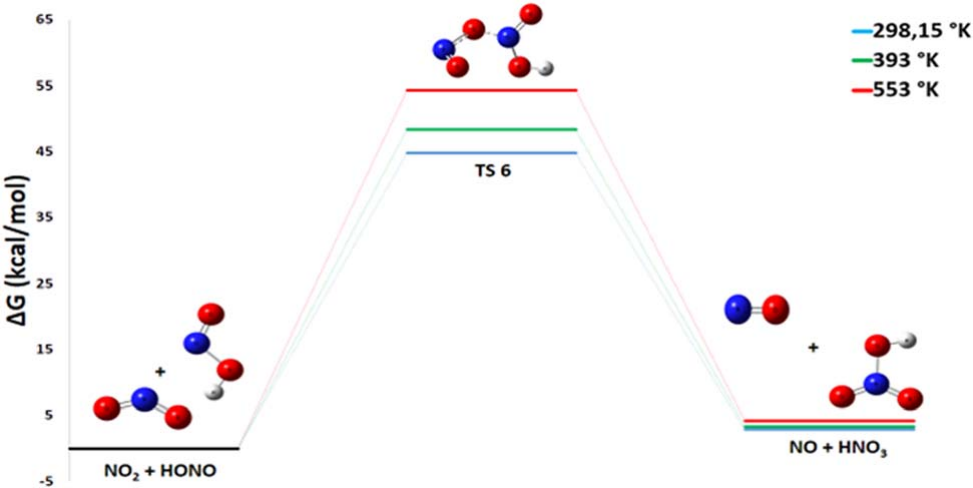
Energy profile of reaction pathway 4 at various temperatures and atmospheric pressure.

The reaction is moderately endothermic and thermodynamically unfavorable over the investigated temperature range. The Gibbs free energy change (Δ*G*°) remains positive, increasing slightly from 2.9 kcal mol^−1^ at 298 K to 4.2 kcal mol^−1^ at 553 K. Similarly, the enthalpy change shows a small variation, rising from 1.2 kcal mol^−1^ at 298 K to 1.3 kcal mol^−1^ at 553 K. Concurrently, the activation energy (*E*_a_) is substantial and rises from 44.8 to 54.3 kcal mol^−1^ (Fig. 14). The combined thermodynamic and kinetic data indicate that this step is both endothermic and characterized by a high-energy barrier. Consequently, Pathway 4 is expected to be slow and is unlikely to compete effectively with more favorable routes, contributing minimally to the overall decomposition mechanism of ADN.

#### 3.3.6 Pathway 5: production of N_2_ and H_2_O

Pathway 5 proceeds through three sequential elementary steps, culminating in the formation of water and inert nitrogen gas. The steps are as follows:

12 NH_2_ + NO → H_2_NNO

**Fig. 15:**
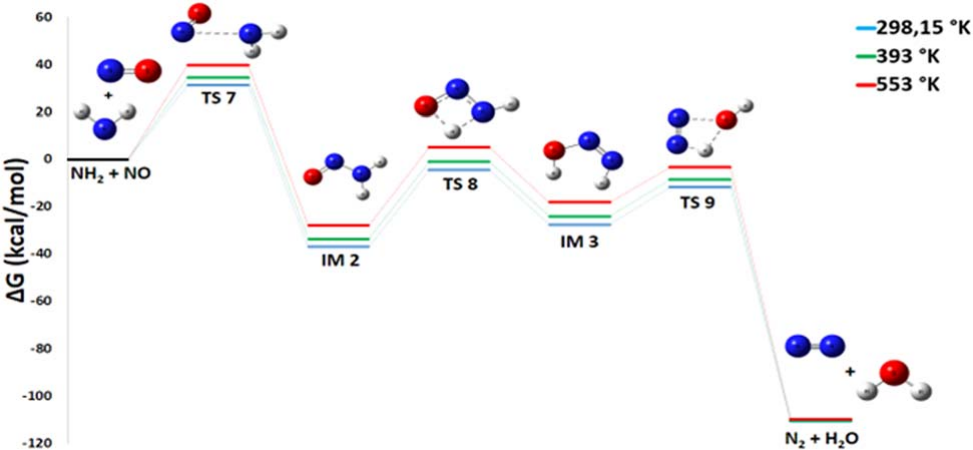
Energy profile of reaction pathway 5 at various temperatures and atmospheric pressure.

This step is exothermic and thermodynamically spontaneous over the investigated temperature range. The Gibbs free energy change (Δ*G*°) varies from −37 to −28 kcal mol^−1^, while the enthalpy change (Δ*H*°) ranges between −47.2 and −75.5 kcal mol^−1^. These negative values confirm the energetic favorability of this transformation. It proceeds with a moderate activation barrier (*E*_a_ between 31 and 39 kcal mol^−1^) Fig. 15. The calculated Δ*G*° becomes less negative with increasing temperature, indicating a slight reduction in thermodynamic driving force at higher temperatures.

13 H_2_NNO → HN_2_OH

This rearrangement is slightly endothermic (Δ*G*° = +9.4 kcal mol^−1^). Our calculations did not identify a distinct transition state for this elementary process, indicating an extremely low or insignificant intrinsic energy barrier.

14 HN_2_OH → N_2_ + H_2_O

This final step is highly exothermic, with Δ*G*° values between −83 and −92 kcal mol^−1^. It has a moderate activation energy (*E*_a_ = 15 kcal mol^−1^, 16 kcal mol^−1^) that decreases slightly with rising temperature.

The pronounced exergonic character of the final step rationalizes the experimental observation of molecular nitrogen formation at elevated temperatures. Indeed, Izato *et al.*^24^ detected N_2_ (alongside N_2_O) during the initial decomposition of ADN. Our computational results suggest that Pathway 5 is a plausible route responsible for this N_2_ production, particularly at higher temperatures where the transformation to N_2_ and H_2_O is most favored.

This study demonstrates that temperature significantly influences the decomposition of ADN, affecting both thermodynamic and kinetic parameters.^31^ These computational findings, consistent with the work of Vyazovkin & Wight^27^ and Izato *et al.*,^24^ confirm that increasing temperature selectively favors decomposition channels yielding gaseous products (N_2_O, NO, N_2_), which are critical for propulsion performance.

### 3.4. Solvent effects on ADN thermal decomposition

This paper examines the solvent variation influence on the decomposition reaction mechanisms. We present a study focused on the impact of two representative solvents: water (H_2_O) and methanol (MeOH). This selection is justified by their potential role in stabilizing key molecular species within aerospace applications.

#### 3.4.1 Initiation step: ADN to HDN and NH_3_

The energy required for the decomposition of ADN is strongly dependent on the surrounding medium's ability to stabilize the resulting species. A key parameter in this process is the solvent's dielectric constant (*ε*). A solvent with a high dielectric constant, such as water (*ε* = 78), significantly attenuates electrostatic interactions between ions, thereby facilitating their separation. Methanol, which also possesses a relatively high dielectric constant (*ε* = 33), exerts a comparable though slightly less pronounced effect. Furthermore, protic solvents like water and methanol can form hydrogen bonds with the ionic species, providing additional stabilization.

**Fig. 16:**
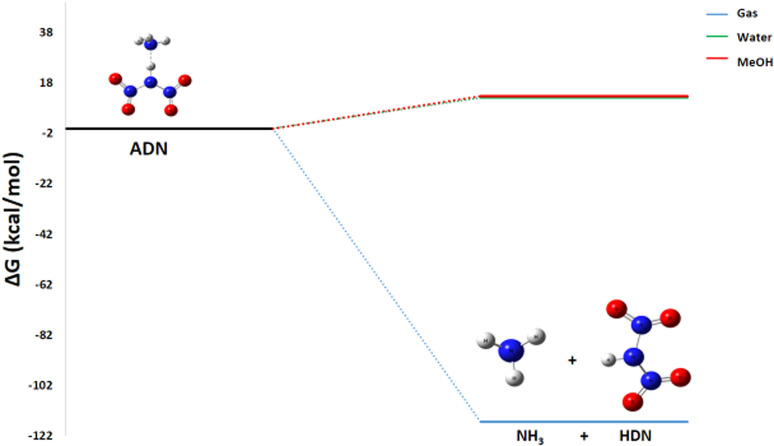
Energy profile of the initiation step in various media.

The calculated Gibbs free energy values and enthalpy changes Table 3, Δ*G*° = 12.2 kcal mol^−1^, Δ*H*° = 19.0 kcal mol^−1^ in water and Δ*G*° = 12.8 kcal mol^−1^ Δ*H*° = 18.8 kcal mol^−1^ in methanol, confirm the non-spontaneous and endothermic nature of this reaction in solution. This result aligns with physical considerations: water, due to its high polarity and remarkable capacity for hydrogen bonding, very effectively stabilizes the constituent ions of solid ADN. Consequently, additional energy (positive Δ*G*) must be supplied to enable their dissociation into NH_3_ and HDN. The observed difference between the two solvents, though small Fig. 16, directly reflects water's superior ability to solvate and stabilize ions compared to methanol, attributable to its higher dielectric constant and more pronounced hydrogen-bonding capability.

**Table 3:** Relative energies and activation energies for decomposition reaction pathways in various media^a^

Pathway	Elementary reactions	Gas			Water			MeOH		
	Energies (kcal mol^−1^)	Δ*G*	Δ*H*	*E*_a_	Δ*G*	Δ*H*	*E*_a_	Δ*G*	Δ*H*	*E*_a_
I.S*	ADN → HDN + NH_3_	−116.3	**−108.2**	TS.F *	12.2	**19.0**	TS.F *	12.8	**18.8**	TS.F *
1	HDN → HN(NO_2_) + NO_2_	21.9	**35.4**	30.0	22.8	**37.8**	29.66	22.8	**37.8**	29.7
	HN(NO_2_) + NO_2_ → NO_3_ + HNNO	−0.7	**0.1**	5.7	−0.4	**−0.5**	5.10	−0.4	**0.5**	5.1
	NO_3_ + HNNO → HNO_3_ + N_2_O	−68.5	**−75.0**	11.8	−71.3	**−76.8**	TS.F *	−71.3	**−76.8**	TS.F *
2	HN(NO_2_) → HONNO	−20.9	**−20.5**	52.1	−21.3	**−21.0**	50.99	−21.3	**−20.9**	51.0
	HONNO → HNO + NO	1.2	**11.9**	9.4	2.3	**13.0**	10.62	2.3	**13.0**	10.6
3	NH_3_ + NO_2_ → HONO + NH_2_	30.6	**30.3**	TS.F *	28.5	**28.1**	TS.F *	28.5	**28.2**	TS.F *
4	HONO + NO_2_ → HNO_3_ + NO	2.9	**1.26**	44.7	2.3	**0.7**	44.51	2.3	**0.7**	44.5
5	NH_2_ + NO → H_2_NNO	−36.9	**−47.2**	31.2	−38.8	**−49.3**	31.06	−38.7	**−49.2**	31.1
	H_2_NNO → HN_2_OH	9.4	**8.89**	32.4	8.5	**8.4**	34.14	8.5	**8.4**	34.1
	HN_2_OH → N_2_ + H_2_O	−83.2	**−73.5**	15.8	−81.7	**−72.0**	19.50	−81.8	**−72.1**	19.4

a TS.F*: TS-free. I.S*: Initiation Step.

#### 3.4.2 Pathway 1: production of N_2_O and HNO_3_

**Fig. 17:**
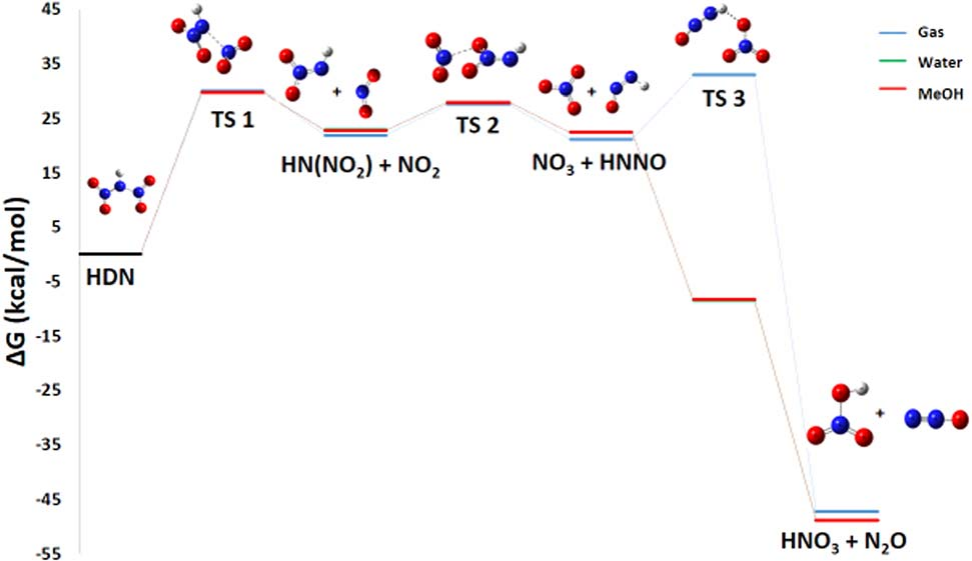
Energy profile of reaction pathway 1 in various media.

In the gas phase, the rate-limiting step is the initial N–NO_2_ bond cleavage in HDN (eqn (15)), which is strongly non-spontaneous (Δ*G*° = 21.89 kcal mol^−1^, Δ*H*° = 35.4 kcal mol^−1^) and slow (*E*_a_ = 30.01 kcal mol^−1^). The presence of a polar protic solvent (water or methanol) alters neither the thermodynamics nor the kinetics of this step significantly Fig. 17.

This indicates that such solvents do not effectively stabilize the highly radical character of the transition state involved in generating two neutral radical species, which are inherently less sensitive to polar solvation effects. The subsequent recombination step (eqn (16)) is fast and slightly favorable, with a Δ*G*° value near zero, indicating a readily reversible equilibrium. Its low activation energy suggests facile radical recombination once the initial barrier is overcome, and solvent effects on this step are minimal. The final step (eqn (17)) is highly exergonic and spontaneous in the gas phase at low temperature Δ*G*° = −68.46 kcal mol^−1^, Δ*H*° = −75.0 kcal mol^−1^ but possesses a moderate kinetic barrier.

In contrast, in aqueous or methanolic solution this step becomes dramatically more favorable due to strong solvation, mainly through hydrogen bonding to the HNO_3_ product, which significantly increases the exergonicity of the reaction. To verify whether this step is truly barrierless in solution, a relaxed scan of the main reaction coordinate was performed, defined as the N–O distance between the NO_3_ radical and the terminal nitrogen atom of HNNO. The scan was carried out with a step size of 0.05 Å, and at each point the electronic energy was calculated while relaxing all remaining geometrical parameters. The resulting energy profile shows a monotonic increase in electronic energy from the reactants toward the products without any local maximum, confirming the absence of an electronic activation barrier for this reaction step. Consequently, this transformation can be considered effectively barrierless under solvated conditions.

15 HN(NO_2_)_2_ → HN(NO_2_) + NO_2_

16 HN(NO_2_) + NO_2_ → NO_3_ + HNNO

17 NO_3_ + HNNO → HNO_3_ + N_2_O

#### 3.4.3 Pathway 2: production of NO and HNO

**Fig. 18:**
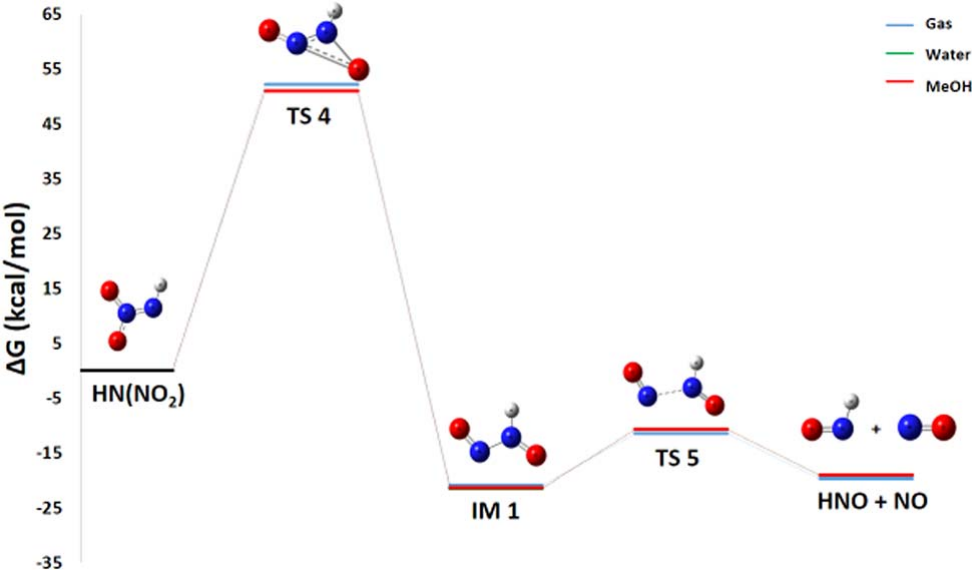
Energy profile of reaction pathway 2 in various media.

This pathway involves an isomerization followed by a fragmentation. A key observation Fig. 18 is that the kinetics (*E*_a_) and thermodynamics (Δ*G*°, Δ*H*°) of these steps are significantly more sensitive to the solvent environment than the initial radical cleavage step discussed previously.

18 HN(NO_2_) → HONNO

19 HONNO → HNO + NO

In the gas phase, this step of Isomerization (eqn (18)) is thermodynamically favorable but kinetically very slow due to an enormous activation barrier. Both water and methanol solvents enhance the thermodynamic favorability (more negative Δ*G*° and Δ*H*°) and slightly reduce the activation energy. This indicates that the solvents interact more favorably with the transition state or the product HONNO than with the reactant HN(NO_2_), likely due to differential stabilization of their respective charge distributions or polarities. In the gas phase, the fragmentation step (eqn (19)) is slightly endergonic yet rapid, characterized by a low activation energy (*E*_a_ = 9.39 kcal mol^−1^), suggesting a reversible equilibrium. In contrast, both solvents exert a net destabilizing effect on this step. They render the reaction less spontaneous (more positive Δ*G*° and Δ*H*°) and slower (higher *E*_a_). This is consistent with polar solvents more effectively stabilizing the reactant HONNO, which is likely more polar than the two neutral radical products (HNO and NO), thereby making its fragmentation more difficult.

The solvent exerts opposing influences on the two steps of this pathway: it slightly facilitates the initial, rate-limiting isomerization but concurrently impedes the subsequent fragmentation.

#### 3.4.4 Pathway 3: production of HONO and NH_2_

**Fig. 19:**
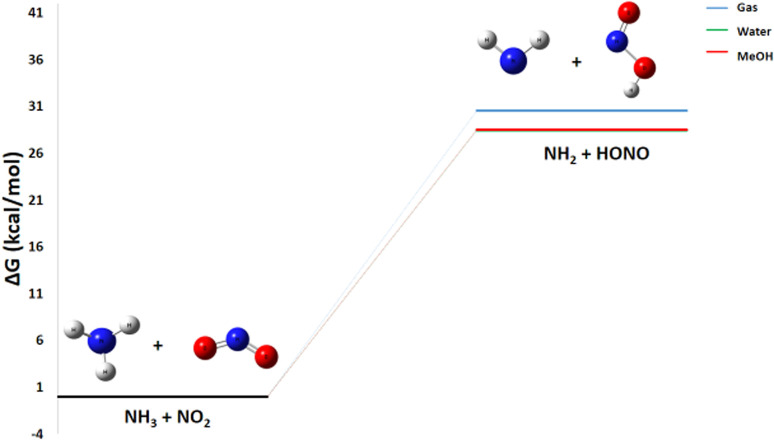
Energy profile of reaction pathway 3 in various media.

This pathway involves the radical-mediated hydrogen abstraction from an ammonia molecule by a nitrogen dioxide radical (NO_2_), yielding nitrous acid (HONO) and NH_2_ radical. This hydrogen transfer reaction has been analyzed in three distinct environments: the gas phase, water, and methanol. In the gas phase, the reaction is strongly endergonic Fig. 19, indicating non-spontaneous behavior where the products (HONO and NH_2_) are significantly less stable than the reactants (NH_3_ and NO_2_). In aqueous and methanolic solutions, the reaction remains endergonic, but to a lesser extent. The decrease in Δ*G*° and Δ*H*° demonstrates that polar solvents provide a slight stabilization of the products relative to the reactants. Although this stabilizing effect is modest, it is measurable. This minor stabilization in solution is chemically consistent. Water and methanol, through their polarity and hydrogen-bonding capacity, can solvate the products HONO and NH_2_ somewhat more effectively than the neutral reactants NH_3_ and NO_2_, thereby marginally reducing the overall energy cost of the reaction.

#### 3.4.5 Pathway 4: production of HNO_3_ and NO

**Fig. 20:**
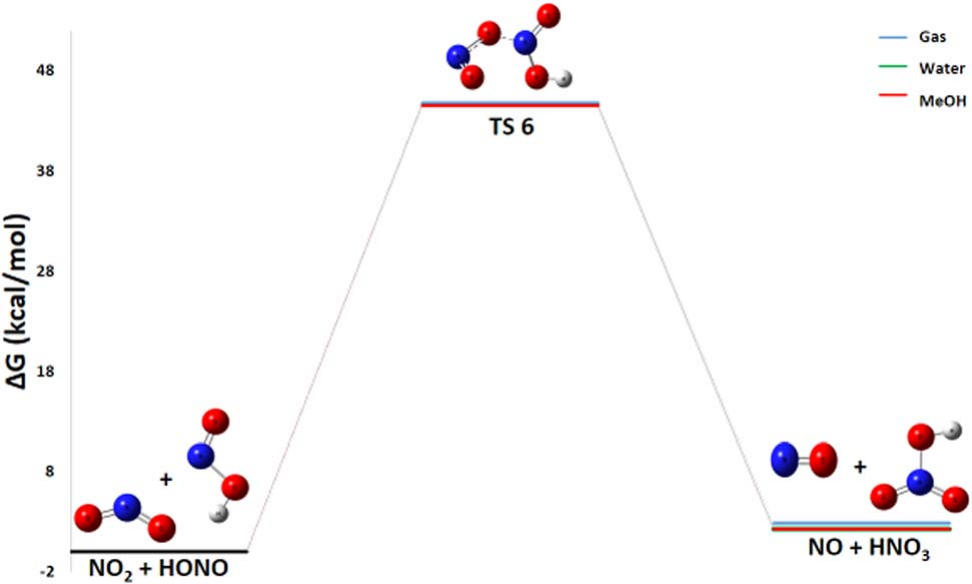
Energy profile of reaction pathway 4 in various media.

The formation of HNO_3_ and NO (eqn (20)) is examined. In the gas phase, this process is slightly endergonic and thus thermodynamically unfavorable. In aqueous solution, it remains unfavorable, although a minor increase in product stability is observed. The behavior in methanol is similar to that in water Fig. 20. Reaction V-6 remains slightly endergonic across all environments. The marginal improvement in solvated media (approximately 0.5 kcal mol^−1^) can be attributed to the more effective solvation of nitric acid (HNO_3_) relative to nitrous acid (HONO), stemming from its greater acidity and enhanced hydrogen-bonding capacity.

20 HONO + NO_2_ → HNO_3_ + NO

Kinetically, the activation barrier remains very high, around 44.5 kcal mol^−1^ in all media. This indicates a highly energetically demanding transition state, likely involving substantial bond reorganization and complex molecular rearrangement. Consequently, the reaction is both thermodynamically unfavorable (Δ*G*° > 0, Δ*H*° > 0) and kinetically very slow (high *E*_a_) in all studied environments. Neither water nor methanol provides significant improvement, suggesting the transition state lacks sufficient polarity to benefit from notable solvation stabilization, and differences in solvation between reactants and products remain minimal.

#### 3.4.6 Pathway 5: production of N_2_ and H_2_O

**Fig. 21:**
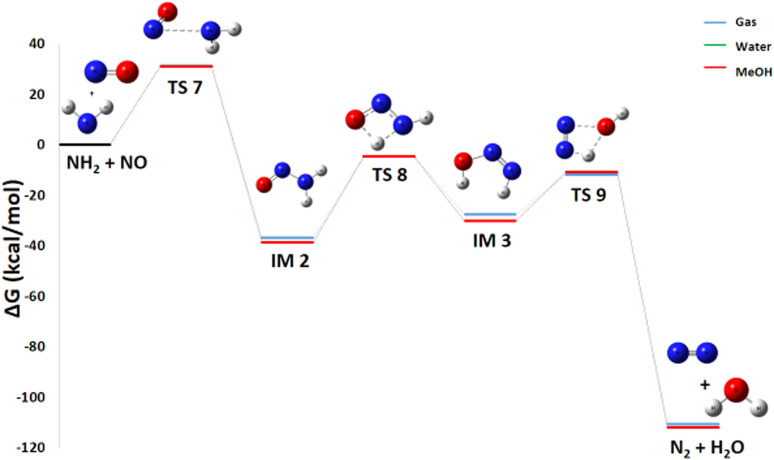
Energy profile of reaction pathway 5 in various media.

The initial step of this pathway (eqn (21)) is highly exergonic but kinetically limited in the gas phase Fig. 21. Polar solvents provide slight stabilization of the products. First, the reaction is strongly exergonic, reflecting the markedly greater stability of intermediate (H_2_NNO) relative to the free radicals. Second, the high activation barrier (31 kcal mol^−1^) is characteristic of radical recombination reactions involving molecular rearrangement. An additional stabilization of approximately 1.7 kcal mol^−1^ is observed in polar media, attributable to favorable electrostatic interactions. The subsequent transformation in step 2 (eqn (22)) is thermodynamically unfavorable and kinetically slow in the gas phase. The presence of solvents further increases the reaction difficulty, slightly raising the activation barrier. In the gas phase, the third step (eqn (23)) is highly exergonic and rapid, as the formation of water and nitrogen constitutes a very favorable process. In solution, the production of water reduces the thermodynamic gain and raises the energy barrier, consistent with solvation stabilization of the formed water molecules.

21 NH_2_ + NO → H_2_NNO

22 H_2_NNO → HN_2_OH

23 HN_2_OH → N_2_ + H_2_O

This study demonstrates that water and methanol, as polar protic solvents, profoundly alter the energy landscape of ADN decomposition compared to the gas phase. Their capacity to form hydrogen bonds and stabilize charged species selectively lowers the energy of certain key steps. This can attenuate activation barriers while at the same time making some initial dissociations thermodynamically less favorable. Water, possessing markedly superior solvation power compared to methanol, further accentuates these effects by levelling the energies of intermediates and promoting the formation of polar products such as HNO_3_. Conversely, the absence of such stabilization in the gas phase results in a steeper energetic profile, where reactivity is heightened but less controlled. Consequently, the solvent emerges as a major determinant of the mechanistic pathway, shaping not only the thermodynamic feasibility but also the positioning of the kinetically limiting steps.

## 4. Conclusion

This theoretical research details the thermal decomposition process using DFT. The process is characterized by a sequence of two distinct phases: first, a rapid initial dissociation into dinitramic acid and ammonia, followed by competing secondary pathways that determine the final distribution of products. The main results show that temperature has a significant impact, specifically stimulating pathways that generate gaseous products essential for propulsion, such as N_2_O, NO, and N_2_. Pathways 1 (leading to N_2_O/HNO_3_) and 5 (leading to N_2_/H_2_O) are identified as predominant, with their kinetics and thermodynamics significantly improved at higher temperatures. On the other hand, the function of polar protic solvents is both complex and selective. They effectively stabilize ions and polar products such as HNO_3_, which increases initial dissociation. However, their impact on steps involving neutral radical intermediates remains limited. These results provide a consistent computational framework that supports laboratory observations, confirming that N_2_O and N_2_ are the primary products of decomposition and clarifying the temporary role of intermediates such as NO_2_ and HNO_3_. This mechanistic understanding highlights the compound's complex reactivity, which balances a large release of energy with decomposition modes that can be adjusted according to the environment and temperature.

## 5. Perspectives

To extend these preliminary findings, forthcoming investigations should address several important directions. Achieving higher chemical precision requires the re-evaluation of key activation barriers using advanced *ab initio* approaches, including CCSD(T) or DLPNO-CCSD(T) methods. Moreover, a deeper exploration of decomposition in the condensed phase—particularly through molecular dynamics simulations incorporating explicit solvent environments—would allow a clearer link between theoretical gas-phase predictions and their practical application in liquid propellant systems. Experimental confirmation of the predicted temperature-dependent branching ratios among pathways 1, 2, and 5 is also essential. This validation could be performed using *in situ* analytical techniques such as coupled TGA-FTIR-MS measurements under controlled thermal conditions. Additionally, investigating how typical propellant constituents, including metals and metal oxides, influence these dominant reaction routes—either by promoting or suppressing them—may provide valuable insights for designing ADN-based propulsion systems with improved stability and controllability.

Finally, a systematic study of non-polar media and ionic liquids as alternative solvent environments may open new perspectives for tuning decomposition behavior. Such efforts could contribute to optimizing reaction kinetics and enhancing the overall performance and reliability of next-generation monopropellants proposed as safer alternatives to hydrazine.

## Author contributions

Conceptualization, Z. Harimech, R. Amrousse and A. Bachar; methodology, Z. Harimech and A. Souagh; software, A. Souagh and M. Salah; validation, Z. Harimech, A. Souagh and A. Mabrouk, resources, R. Amrousse; data curation, Z. Harimech and A. Souagh; writing—original draft preparation, Z. Harimech, writing—review and editing, Z. Harimech, R. Amrousse, K. Toshtaty and S. Azat; visualization, S. Azat and A. Mabrouk; supervision, M. Salah and R. Amrousse.

## Conflicts of interest

There are no conflicts to declare.

## Abbreviations

ADN: Ammonium dinitramide
HDN: Dinitraminic acid
AN: Ammonium nitrate
DFT: Density functional theory
TOF: Time-of-flight
TG-DTA: Thermogravimetry-differential thermal analysis
MS: Mass spectrometry
IR: Infrared spectroscopy
FTIR: Fourier-transform infrared spectroscopy
GC: Gas chromatography
*E*_a_
: Energy activation
Δ*G*: Gibbs free energy
Δ*S*: Entropy
IM: Intermediate
TSF: Transition state free
IS: Initiation step

## Data Availability

The data cannot be shared publicly on the Internet due to the confidentiality of the research project. However, the data are available upon request from the author.
